# hsa-miR-9-5p-Mediated TSPAN9 Downregulation Is Positively Related to Both Poor Hepatocellular Carcinoma Prognosis and the Tumor Immune Infiltration

**DOI:** 10.1155/2022/9051229

**Published:** 2022-05-12

**Authors:** Shengkui Tan, Xin Song, Chenchen Zhang, Yishu Sun, Jiaxi Zhang, Zhongqi Zhang, Rongcheng Zhang, Tianmiao Zhang, Xiaonian Zhu, Hongzhuan Tan

**Affiliations:** ^1^Department of Epidemiology and Health Statistics, Xiangya School of Public Health, Central South University, Changsha, 410078 Hunan, China; ^2^Department of Epidemiology and Health Statistics, Guilin Medical University, Guilin, 541004 Guangxi, China

## Abstract

Tetraspanins (TSPANs) play crucial roles in cell adhesion, migration, and metastasis of human cancer. However, there is no study in revealing the aspects of TSPAN9 traits and its functions in hepatocellular carcinoma (HCC) prognosis. Our study is the first to portray the TSPAN9 expression in HCC tissues with immunohistochemistry (IHC) analysis. Subsequently, a series of bioinformatics analyses such as expression estimation, survival assessment, and correlation analysis were implemented to dig out the possible upstream noncoding RNAs (ncRNAs) for TSPAN9 in HCC. In this way, the relevance within TSPAN9 and tumor immunity was then explored. We found that the TSPAN9 was downregulated in HCC tissues and had a correlation with HCC prognosis. Furthermore, we identified that the AL139383.1-hsa-miR-9-5p axis was the upstream ncRNA-related pathway most associated with TSPAN9 in HCC. Besides that, expression of TSPAN9 held a significantly negative correlation with tumor immunocyte infiltration as well as immune checkpoint CTLA4. TSPAN9-related immunomodulators were mainly enriched in T cell activation, leukocyte cell-cell adhesion, regulation of T cell activation, and regulation of leukocyte cell-cell adhesion signaling pathway. In conclusion, our results indicated that hsa-miR-9-5p-mediated downregulation of TSPAN9 was associated with poor HCC prognosis, immune-related signaling pathway, and tumor immune infiltration.

## 1. Introduction

Liver cancer ranks the fourth cancer death cause with its high mortality worldwide [1]. Among the five deadly cancers in the US, liver cancer is the only cancer with an increasing incidence year by year [2]. Liver cancer has a high prevalence in developing countries and regions [3], especially in Guangxi, China [4]. Hepatocellular carcinoma (HCC) is the most common subtype in primary liver cancer, making up for 75% to 85% of the total liver cancer cases [1]. The primary threat factors for HCC are chronic infection with hepatitis B virus (HBV) [5] or hepatitis C virus (HCV) [6], aflatoxin-contaminated foodstuffs [7], heavy alcohol intake [8], and obesity [9]. In the last few years, the HCC research has created a lot of major breakthrough [6], and even immunotherapy has gradually increased [10]. However, the prognosis of patients with HCC is still not ideal, and the research on biomarkers of HCC has a long way to go [11]. So, we need to find the anticipated target to diagnose and treatment HCC early.

Tetraspanins (TSPANs), also known as the transmembrane 4 superfamily (TM4SF), are small transmembrane proteins. The name of this family refers to four transmembrane fragments and two extracellular rings with a very conservative topology on their sides [12]. TSPANs are main organizers of the cell membrane due to their form so-called tetraspanin-enriched microdomains (TEMs), which usually includes several different tetraspanins along with proteases, immunoglobulin, integrins, and/or other cell-specific receptors. According to the cell type and the molecular type of assembly, TSPANs are involved in cell migration, cell-cell signal transduction, immune response, tumor progression, and metastasis [13]. Studies have shown that four transmembrane proteins can indirectly regulate metastasis by mediating cell interactions in the immune system through exosomes [14]. More importantly, targeting a single tetraspanin with antibodies could affect tumor progression [14]. It is well known that TSPANs could interact with a variety of key white blood cell proteins, including integrins, signaling molecules, and immune receptors. These tetraspanin-chaperone protein interactions control several basic cellular processes, such as movement, antigen presentation, proliferation, and antibody production in immunocytes [15]. Research evidence indicates that tetraspanin in B lymphocytes is important for antibody production [16]. For example, the interaction between tetraspanin CD81 and B-cell receptor (BCR) complex is very important for the expression of CD19 and IgG production, but tetraspanin CD37 hinders the production of IgA and is important to the production of IgG [17]. In addition, CD9 and CD151 tetraspanins accumulate on the T cell side of immune synapses and support integrin mediated signal transduction to immune synapses [18]. TSPANs may promote or inhibit tumor invasion and metastasis through changes in movement and mitotic behavior and/or tumor cell microenvironment interaction [19]. As a regulator of cell membrane structure and function, TSPANs have become a diagnostic and prognostic marker and therapeutic target for tumor progression.

In this study, we first analyzed the clinical significance of the differentially expressed TSPAN9 protein in HCC tissues by immunohistochemistry (IHC) and validated it in online databases. Then, we conducted our study on all patterns of noncoding RNA (ncRNA) regulation to TSPAN9 in HCC. At last, we identified the relationship between the expression of TSPAN9 with immunocyte infiltration and immune checkpoint in HCC. Our results showed that the patients with ncRNAs mediated TSPAN9 downregulation had a poor prognosis and tumor immune infiltration.

## 2. Materials and Methods

### 2.1. Tissue Samples

Ninety pairs of HCC and adjacent tissues were taken from the First Affiliated Hospital of Guilin Medical University and Municipal People's Hospital Affiliated to Guilin Medical University from 2014 to 2018. All patients were diagnosed as HCC through pathology and did not receive treatment before operation. These patients had complete clinical data, including gender, age, tumor size, pathological grade, cirrhotic nodules, clinical stage, HBV infection, and serum AFP. All HCC cases were followed up by regularly examination or phone tracking. The survival time was calculated from the first day after operation until death, lost from observation, tumor recurrence, or metastasis. Each patient participating in the study signed informed consent. Our study was allowed by the Ethics Committee of Guilin Medical University (GLMC2015004).

### 2.2. IHC

The Xinchao Company (Shanghai, China) produced sample tissues into tissue chip. Then, the expression of TSPAN9 was detected by IHC kit (Maixin Biotechnologies, Fuzhou, China). The specific steps are consistent with the previous research [20]. All IHC sections were examined blindly. The expression level of TSPAN9 in all samples was evaluated as follows. (1) Five random visual fields of each tissue were analyzed under a 400× microscope. The number of positive cells and the total number of cells in each visual field were calculated to obtain the positive cell rate. The score for the rate of positive cell: 0 was ≤5%, 1 was 6-25%, 2 was 26-50%, 3 was 51-75%, and 4 was >75%. (2) The score based on dyeing strength: 0 for uncolored, 1 for light yellow, 2 for yellow brown, and 3 for brown. In the end, multiply the two scores obtained before, 0 for (-), 1-4 for (+), 5-8 for (++), and 9-12 for (+++). The positive of TSPAN9 expression was defined as >4, and the negative expression was defined as ≤4.

### 2.3. Kaplan-Meier Analysis

The Kaplan-Meier (https://kmplot.com) with access to the survival effects of genes or miRNAs on more than 20 cancers including HCC was used to analyze the survival rates of TSPAN9 and hsa-miR-9-5p in HCC [21]. The results of *P* < 0.05 were considered statistically significant.

### 2.4. Candidate miRNA Prediction

Multiple target gene prediction programs were used to predict the upstream miRNAs of TSPAN9, consisting of miRanda, PITA, PicTar, RNA22 [22], miRmap [23], microT [24], and TargetScan [25]. Only the candidate miRNAs appeared common to the above three or more programs were included in the subsequent analysis.

### 2.5. StarBase (ENCORI) Database Analysis

StarBase, also known as ENCORI, is a database focusing mostly on ncRNA interactions [26]. ENCORI was used to conduct analysis of expression correlation between miRNAs and TSPAN9, lncRNAs and hsa-miR-9-5p, or lncRNAs and TSPAN9 in HCC. In addition, StarBase was introduced for the prediction on the candidate lncRNAs possibly binding to hsa-mir-9-5p.

### 2.6. TCGA Database Analysis

The data of candidate ncRNAs were downloaded from TCGA database. Furthermore, these data were standardized, and then, the differential expression and prognosis were analyzed by R software package limma and survival [27]. *P* < 0.05 was regarded as statistically significant.

### 2.7. TIMER Database Analysis

The Tumor Immune Evaluation Resource (TIMER) is a comprehensive resource for correlation analysis of immunocyte infiltration in various cancer types [28]. It was adopted to reveal the association between the level of TSPAN9 expression with the level of immunocyte infiltration and the level of immune checkpoint expression in HCC. *P* < 0.05 was regarded to have significant difference.

### 2.8. Immunomodulators Associated with TSPAN9 Analysis

The immune modulators related to TSPAN9 were searched from the comprehensive database TISIDB for elucidating tumor immune system interrelations [29]. The portal is based on the merged from the other data, such as TCGA, high-throughput screening data, exome and RNA sequencing data, PubMed database, and other public databases. We selected immunosuppressive and immunostimulatory agents that were significantly associated with TSPAN9 gene expression (Spearman's correlation test, *P* < 0.05). Next, we performed GO annotation and KEGG pathway enrichment analyses of the resulting protein networks.

### 2.9. Statistical Analysis

Patient clinical data were statistically analyzed by SPSS 19.0 software. McNemar *χ*^2^ test was introduced to analyze the expression difference of TSPAN9 in HCC and adjacent tissues. Other statistical analyses of this study were calculated based on the above online database. The threshold of statistical significance was *P* < 0.05.

## 3. Results

### 3.1. Relationship within TSPAN9 Expression and HCC Prognosis

**Figure 1:**
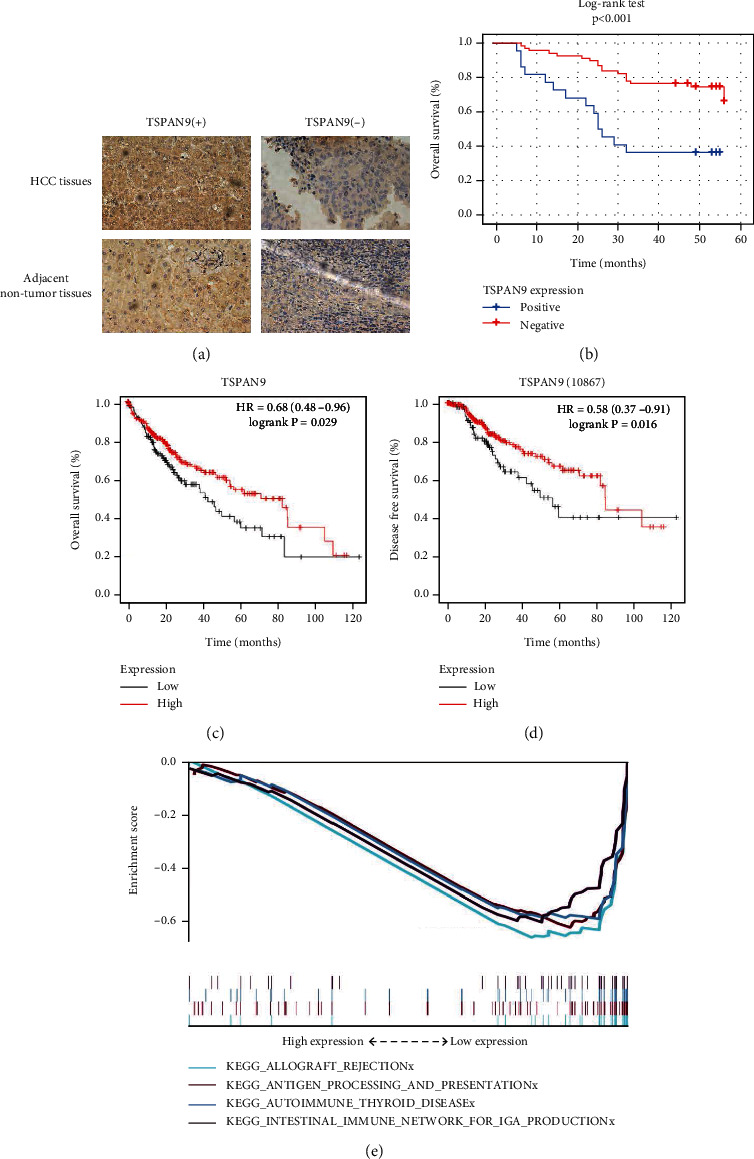
The correlation between the expression of TSPAN9 and HCC prognosis. (a) Representative results of immunohistochemical staining. (b) Log-rank test of survival time in patients with HCC. (c and d) The Kaplan-Meier plotter on the analysis to OS (c) and DFS (d) of TSPAN9 in HCC patients. (e) In HCC, the enrichment of pathways negatively correlated with the expression level of TSPAN9.

Firstly, we explored the expression of TSPAN9 in HCC tissues by IHC staining. We observed that the TSPAN9 expression in HCC tissues (66/90) was notably lower than the TSPAN9 expression in the adjacent nontumor liver tissues (87/90) (Figure 1(a) and Table 1, *P* < 0.001). Besides, the correlation between TSPAN9 expression and patient-related clinicopathological factors was analyzed. The results indicated that TSPAN9 expression was correlated with age, recurrence, and tumor size (Table 2, *P* < 0.05), but had no relationship with gender, pathological grade, number of tumors, cirrhotic nodules, or tumor capsule of HCC patients. Log-rank test found that the survival time of HCC patients with positive TSPAN9 expression was longer than that of HCC patients with negative TSPAN9 expression. With the intention of further verifying the prognostic value of TSPAN9 in HCC, we exploited the Kaplan-Meier model for the analysis toward the effect of TSPAN9 expression on the prognosis of HCC patients. And it turned out that the overall survival (OS) and disease-free survival (DFS) of HCC patients with positive expression of TSPAN9 were both significantly higher than that of HCC patients with negative expression of TSPAN9 (Figures 1(c) and 1(d)). Multivariate COX analysis revealed that TSPAN9 was an independent protective factor for the patients with HCC (Table 3). All the results implied that the TSPAN9 expression has prognostic value for HCC patients.

To explore the potential role of TSPAN9 in HCC, we performed KEGG pathway enrichment analysis, and the results unveiled that downregulation of TSPAN9 was correlated to multiple immune and cancer pathways (Figure 1(e)), including antigen processing and presentation, intestinal immune network for IgA production, autoimmune thyroid disease, and allograft rejection signaling pathway.

### 3.2. Prediction of Upstream ncRNAs of TSPAN9

**Figure 2:**
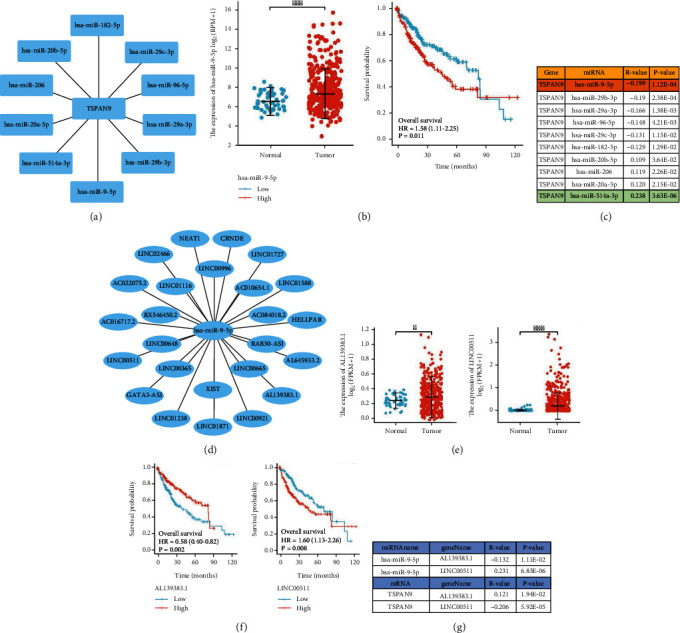
Identification of the upstream noncoding RNA axis of TSPAN9 mRNA in HCC. (a) TSPAN9 upstream miRNA regulatory network. (b) The TCGA database analysis on has-miR-9-5p in HCC expression and the prognostic value. (c) The expression correlation within those predicted miRNAs and TSPAN9 in HCC. (d) has-miR-9-5pupstream lncRNA regulatory network. (e) Two upstream lncRNAs of has-miR-9-5p high expressed in hepatocellular carcinoma. (f) Survival curves of these two meaningful lncRNAs. (g) Correlation analysis between these two meaningful lncRNAs, has-miR-9-5p, and TSPAN9.

In gene expression regulation, the role of ncRNAs has been widely recognized. To determine whether TSPAN9 was regulated by ncRNAs, firstly, we predicted upstream miRNAs that may bind to TSPAN9, and then, we found 10 miRNAs. In addition, we used Cytoscape software to visualize this result and established a miRNA-TSPAN9 regulatory network (Figure 2(a)). From the mechanism of miRNA regulating target gene expression, candidate miRNAs were negatively correlated with TSPAN9. Thus, as shown in Figure 2(c), the expression of TSPAN9 was significantly negatively related to hsa-miR-9-5p in HCC. Therefore, we selected hsa-miR-9-5p as the most potential upstream miRNA and performed expression and prognostic analysis on it. The results showed that hsa-miR-9-5p was significantly upregulated in patients with HCC, which was related to the poor prognosis of patients with HCC (Figure 2(b)). All these results indicated that hsa-miR-9-5p can be the most potential upstream regulatory miRNA of TSPAN9 in HCC. Moreover, we used the ENCORI database to identify the upstream candidate long-chain noncoding RNAs (lncRNAs) of hsa-miR-9-5p and found 25 candidate lncRNAs in HCC (Figure 2(d)). According to the competing endogenous RNA (ceRNA) hypothesis, lncRNA could increase mRNA expression by competitively binding to shared miRNAs. Therefore, there should be negative correlation between lncRNA and miRNA or positive correlation between lncRNA and mRNA. Through expression, survival, and correlation analyses, we screened out that AL1393838.1 might be the most potential upstream lncRNA for hsa-miR-9-5p (Figures 2(e)–2(g)). Therefore, we speculate that AL1393838.1 could increase TSPAN9 expression by competitively binding to shared hsa-miR-9-5p, and AL1393838.1/hsa-miR-9-5p/TSPAN9 axis is a key player in HCC.

### 3.3. TSPAN9 Negatively Correlates with Infiltration of Immunocytes and Expression of Immune Checkpoint in HCC

**Figure 3:**
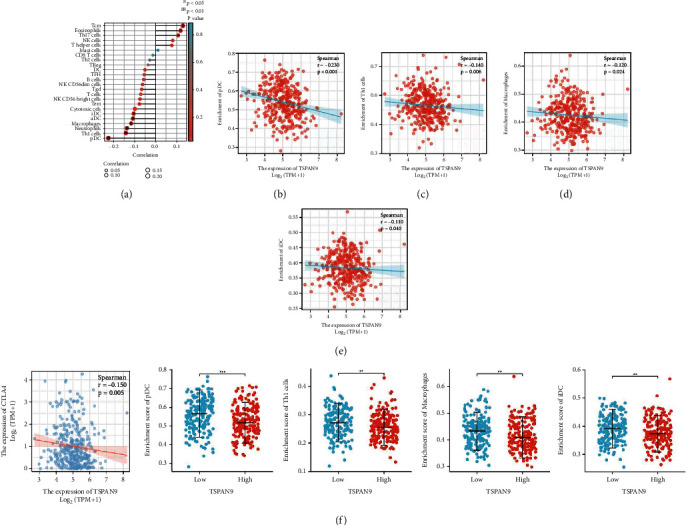
The TSPAN9 correlation analysis on the indexes of immune cell infiltration and immune checkpoint. (a) A lollipop diagram on the correlation and the correlation degree of TSPAN9 and the multiple immune cell infiltration in HCC. (b–e) The correlation of TSPAN9 with pDC (b), Th1 cell (c), macrophage (d), and iDC (e) infiltration level. (f) The expression correlation of TSPAN9 and CTLA4.

TSPAN9 is a member of TSPANs and participates in the formation of TEMs, which play a key role in the immune microenvironment [15]. Therefore, the association between TSPAN9 expression and the immunocyte infiltration degree was revealed in HCC. As shown in Figure 3(a), the expression of TSPAN9 was notably and negatively associated with the infiltration level of plasmacytoid predendritic cells (pDC), T helper 1 (Th1) cells, neutrophils, macrophages, activated-dendritic cells (aDC), and immature dendritic cells (iDC). The specific *P* value and correlation coefficient were shown in Figures 3(b)–3(e). Finally, we figured out the correlation between TSPAN9 expression and the three most common immune checkpoints (PD-1, PD-L1, and CTLA4) in HCC and observed significant negative relationship within the expressions of TSPAN9 and CTLA4 (Figure 3(f)).

### 3.4. TSPAN9-Related Immunomodulators and Their Pathway Enrichment in HCC

**Figure 4:**
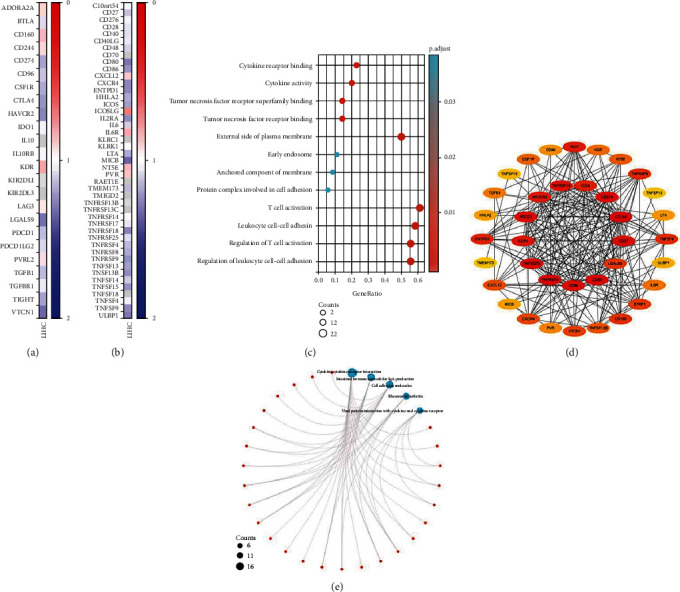
TSPAN9-related immunomodulators and their pathway enrichment in HCC. (a) Heat map of the correlation between HCC immunosuppressants and TSPAN9. (b) The relevance heat map on HCC immunostimulants and TSPAN9. (c) The GEO pathway enrichment analysis of immunomodulators related to TSPAN9 in HCC. (d) Protein-protein network of TSPAN9-associated immunomodulatory closely related genes in HCC. (e) The KEGG pathway enrichment analysis of immunomodulators related to TSPAN9 in HCC.

Finally, in order to further explore the potential of TSPAN9 in immune system, we screened the immunomodulators associated with TSPAN9 and obtained 12 immunosuppressive factors and 25 immunostimulatory factors associated with TSPAN9 (Figures 4(a) and 4(b)). Pathway enrichment analysis of GEO found that these 37 immunoregulatory factors related to TSPAN9 were mainly enriched in T cell activation, external side of plasma membrane, regulation of T cell activation, leukocyte cell-cell adhesion, and regulation of leukocyte cell-cell adhesion signaling pathway. Then, we constructed the PPI network of these 37 genes (Figure 4(d)). At last, it was revealed by a KEGG and GO pathway enrichment analyses that there were 37 genes mainly enriched in the aspects of cell adhesion molecules, cytokine-cytokine receptor interaction, intestinal immune network for IgA production, rheumatoid arthritis, and viral protein interaction with cytokine and cytokine receptor signaling pathway.

## 4. Discussion

On the basis of clinical practice, prognosis of HCC patients nowadays remains extremely unoptimistic. Accumulating evidence shows that TSPANs are the indispensable players in the initiation and progression of various human cancers [30]. TSPAN9 is a crucial player in HCC development, but the study on it is still vacant and needs exploring.

Our study began with the expression and prognosis analyses towards TSPAN9 via IHC staining and Log-rank test and ended up with the Kaplan-Meier plotter analysis for the verification on the TSPAN9 prognostic effect. The results of IHC indicated that the TSPAN9 expression in HCC tissues was notably lower than that in the adjacent nontumor liver tissues. The survival analysis indicated that the HCC patients with low TSPAN9 expression had poor prognosis. Qi et al. suggested that TSPAN9 could inhibit cell migration of gastric cancer. Moreover, EMILIN1 could promote the tumor-suppressive effect of TSPAN9 [31]. This report was consistent with the tumor suppressor role of TSPAN9 in HCC shown in our results. Then, in the purpose of digging out the underlying mechanism of TSPAN9 in HCC, we further explored the signal pathways associated with low expression of TSPAN9. The KEGG findings revealed that low TSPAN9 expression was closely associated with the enrichment of immune pathways.

Interaction within ncRNAs (microRNAs, lncRNAs, and circular RNAs) in posttranscription is generally portrayed as that they participate in the gene expression regulation with each other in a manner of mutual endogenous competition [32–34]. Our analyses on expression, correlation, and survival showed that hsa-miR-9-5p was the optimal miRNA in the upstream of TSPAN9 in HCC. It promotes HCC cell proliferation, migration, and invasion in a way of targeting ESR1 [35], Klf4 [36], and SOX11 [37]. These results met with our expectations; thus, we speculated that the upregulation of hsa-miR-9-5p in HCC was responsible for TSPAN9 downregulation. Hence, the potential upstream lncRNAs of the hsa-miR-9-5p/TSPAN9 axis could be the tumor-suppressor lncRNAs in HCC. By performing expression, survival, and correlation analysis, the most potential lncRNA, AL139383.1, was identified. Although there had not been any reports on AL139383.1 research, our study revealed that HCC patients with high AL139383.1 expression had better prognosis than those with low AL139383.1 expression, suggesting its tumor suppressor function in this course. Taken together, AL139383.1/hsa-miR-9-5p/TSPAN9 axis was considered as a potential regulatory pathway in HCC.

It has been proved that the infiltration of tumor immune cells is a possible factor for the efficacy of radiotherapy, chemotherapy, and immunotherapy [38–40]. Our study showed that TSPAN9 was negatively and significantly related with different immunocytes infiltration, including pDC, Th1 cells, macrophages, neutrophils, aDC, and iDC in HCC. The strongest negative correlation with TSPAN9 expression level was pDC, with a correlation coefficient of 0.23. As a special DC population, pDC is more possible to generate massive type I interferon (IFN), and its unique capabilities are the merits to contribute to the link within innate immunity and adaptive immunity [41, 42]. Since the association of pDC with viral infections, maybe we can associate pDC with HBV in HCC through TSPAN9.

In addition, the full expression of immune checkpoints is also one of the key factors for the immunotherapy therapeutic effect [43]. In this context, our study has made a fully demonstration to the correlation between TSPAN9 and the 3 most common immune checkpoints (PD1, PD-L1, and CTLA-4). Our results revealed that low TSPAN9 expression had a significant correlation with high level of CTLA-4 in HCC, implying that highly expressed TSPAN9 may indicate better immunotherapy efficacy.

In sum, firstly, we found that the TSPAN9 was downregulated in HCC tissues, and high expression of TSPAN9 in HCC implied a good prognosis for HCC patients. Secondly, the axis of AL139383.1-hsa-miR-9-5p was proved to be the most possible upstream regulatory mechanism of TSPAN9 in HCC. Thirdly, our results suggested that TSPAN9 may play its tumor-suppressor role by reducing tumor immunocyte infiltration, enrichment of immune-related signaling pathway, and expression of immune checkpoint proteins in HCC patients. TSPAN9 may be used as a predictor of the prognosis and the efficacy of CTLA4 immunosuppressive agents in HCC patients. Nevertheless, these findings ought to be further verified by more fundamental functional experiments and large-sample clinical cohort trials.

## Figures and Tables

**Table 1:** TSPAN9 expression in paired HCC tissues and adjacent nontumor tissues.

HCC tissues	Adjacent nontumor tissues		Total
	Positive	Negative	
Positive	64	2	66
Negative	23	1	24
Total	87	3	90

Notes: *P* < 0.001. *P* value is based on the McNemar *χ*^2^ tests. HCC: hepatocellular carcinoma.

**Table 2:** Association between the expression of TSPAN9 and clinicopathological features of HCC patients.

Variables	Total	TSPAN9 staining		*χ* ^2^	*P*
		Positive	Negative		
Age					
<50	37	32	5	4.065	**0.044**
≥50	53	36	17		
Gender					
Male	10	6	4	1.474	0.225
Female	80	62	18		
Recurrence					
No	41	36	5	6.118	**0.013**
Yes	49	32	17		
Tumor size					
<5	55	46	9	5.000	**0.025**
≥5	13	22	35		
Pathological grade					
I+II	43	33	10	0.063	0.802
III	12	35	47		
Number of tumors					
<2	79	60	19	0.054	0.816
≥2	11	8	3		
Cirrhotic nodules					
<3	56	42	14	0.025	0.875
≥3	34	26	8		
Tumor capsule					
Complete	43	34	9	0.551	0.458
Incomplete	47	34	13		
Clinical stage					
I	63	50	13	1.650	0.199
II+III	27	18	9		
Total bilirubin (*μ*mol/L)					
<17	65	47	18	2.244	0.134
≥17	24	21	3		
Hepatitis B virus					
Positive	19	13	6	0.854	0.355
Negative	70	55	15		
Serum AFP (ng/mL)					
<25	37	31	6	2.303	0.129
≥25	53	37	16		
ALT (ng/mL)					
<40	52	36	16	2.668	0.102
≥40	38	32	6		
GGT (U/L)					
<50	45	36	9	0.963	0.327
≥50	45	32	13		

Notes: bold values indicate significance.

**Table 3:** COX regression analysis for overall survival of HCC patients after surgery.

Variables	B	Wald	*P* value	OR	95% CI	
					Lower	Upper
TSPAN9	-0.962	6.322	**0.012**	0.382	0.181	0.809

Notes: bold values indicate significance.

## Data Availability

The data used to support the findings of this study are included within the article.
